# Phosphorous Supplementation Alleviates Drought-Induced Physio-Biochemical Damages in *Calligonum mongolicum*

**DOI:** 10.3390/plants11223054

**Published:** 2022-11-11

**Authors:** Abd Ullah, Akash Tariq, Fanjiang Zeng, Jordi Sardans, Corina Graciano, Sami Ullah, Xutian Chai, Zhihao Zhang, Maierdang Keyimu, Muhammad Ahsan Asghar, Hafiz Hassan Javed, Josep Peñuelas

**Affiliations:** 1 Xinjiang Key Desert Plant Roots Ecology and Vegetation Restoration Laboratory, Xinjiang Institute of Ecology and Geography, Chinese Academy of Sciences, Urumqi 830000, China; 2 State Key Laboratory of Desert and Oasis Ecology, Xinjiang Institute of Ecology and Geography, Chinese Academy of Sciences, Urumqi 830000, China; 3 Cele National Station of Observation and Research for Desert-Grassland Ecosystems, Cele 848300, China; 4 University of Chinese Academy of Sciences, Beijing 100045, China; 5 CSIC, Global Ecology Unit, CREAF-CSIC-UAB, Bellaterra, 08193 Barcelona, Catalonia, Spain; 6 CREAF, 08193 Cerdanyola del Vallès, Catalonia, Spain; 7 Instituto de Fisiología Vegetal, Consejo Nacional de Investigaciones Científicas y Técnicas, Universidad Nacional de La Plata, Buenos Aires B1406, Argentina; 8 Department of Botany, University of Peshawar, Peshawar 25000, Pakistan; 9 Department of Biological Resources, Agricultural Institute, Centre for Agricultural Research, ELKH, 2462 Martonvásár, Hungary; 10 College of Agronomy, Sichuan Agricultural University, Chengdu 611130, China

**Keywords:** hyper-aridity, drought, antioxidant mechanism, N metabolism, P fertilization, xerophytes

## Abstract

*Calligonum mongolicum* is a phreatophyte playing an important role in sand dune fixation, but little is known about its responses to drought and P fertilization. In the present study, we performed a pot experiment to investigate the effects of P fertilization under drought or well-watered conditions on multiple morpho-physio-biochemical attributes of *C. mongolicum* seedlings. Drought stress leads to a higher production of hydrogen peroxide (H_2_O_2_) and malondialdehyde (MDA), leading to impaired growth and metabolism. However, *C. mongolicum* exhibited effective drought tolerance strategies, including a higher accumulation of soluble sugars, starch, soluble protein, proline, and significantly higheractivities of peroxidase (POD) and catalase (CAT) enzymes. P fertilization increased the productivity of drought-stressed seedlings by increasing their growth, assimilative shoots relative water content, photosynthetic pigments, osmolytes accumulation, mineral nutrition, N assimilation, and reduced lipid peroxidation. Our findings suggest the presence of soil high P depletion and *C. mongolicum* high P requirements during the initial growth stage. Thus, P can be utilized as a fertilizer to enhance the growth and productivity of *Calligonum* vegetation and to reduce the fragility of the hyper-arid desert of Taklamakan in the context of future climate change.

## 1. Introduction

The drylands have enormous socioeconomic and scientific values. For generations, arid regions have provided livelihoods to dependent communities through their biological and cultural diversity [1]. Despite this, global awareness of the importance of arid regions, and the effort required to manage and protect them, remains low [2,3]. Arid lands suffer irreparable damage due to biodiversity loss, unsustainable use, rigorous agricultural expansion, and global climate change [4]. As a result of such degradation, ground-water resources, valuable plant species, ecosystem productivity, and communities dependent on these systems may be severely affected. Arid lands host ecosystems that are particularly susceptible to climate change and unsustainable human activities [5]; hence, understanding their importance, threats to their biodiversity and productivity, and sustainable management is crucial to meeting global biodiversity conservation targets. Many arid and semi-arid regions of the world have been adversely affected by desertification [6]. Soil water and nutrients are closely related to each other, affecting plants’ development, growth, and productivity. These two groundwater components are essential for establishing and maintaining xerophytes [7]. Xerophytes refer to plant species that can survive prolonged periods of drought. They must extend their roots rapidly to acquire groundwater resources and reduce the threat of water deficit and nutrient limitation to adapt to hyper-arid desert conditions. However, juvenile seedlings are particularly vulnerable to water deficit conditions [8]. Generally, hyper-arid ecosystems are characterized by shallow water and extreme nutrient limitations (N & P), making spontaneous seedling establishment difficult for xerophytes. They, therefore, threaten natural vegetation renewal. Therefore, it is critical to investigate the response of young xerophytes to water and nutrient availability before their roots acquire groundwater resources. Water and nutrient availability can affect plant morphological and physio-biochemical metabolism. For instance, a lack of soil water and nutrients diminishes the process of photosynthesis via stomatal closure or metabolic changes. This leads to more excessive light energy absorption than the utilization of the photosynthetic apparatus, causing photoinhibition and photo-oxidative stress due to the excessive accumulation of reactive oxygen species (ROS), including hydrogen peroxide (H_2_O_2_) and superoxide anion (O_2_^−^) [9]. 

In response, plants have developed various protective mechanisms to counterbalance excess ROS production and prevent damage to photosynthetic machinery. These mechanisms involve alterations in light-harvesting antennae, the activation of antioxidant enzymatic activities (peroxidase [POD], catalase [CAT], superoxide dismutase [SOD], glutathione reductase [GR], etc.), and antioxidant metabolites (carotenoids, glutathione, ascorbate) [9]. Plants can accumulate compatible solutes (i.e., soluble sugars, proline) under drought stress to maintain their cell turgor, gas exchange capabilities, and growth [10]. Plant cells require large amounts of nitrogen (N), a key component of several biomolecules, including proteins, amino acids, nucleic acids, and other important metabolites. N is primarily absorbed by roots in the form of nitrate (NO_3_^−^), which is transported to leaves for assimilation [11]. The enzyme nitrate reductase (NR) in the cytoplasm reduces NO_3_^−^ to NO_2_^−^, which is then transported to chloroplasts for assimilation into NH_4_^+^ by the action of nitrite reductase (NiR) [12]. The NH_4_^+^ assimilation into glutamine and subsequently into glutamate is carried out by glutamine synthetase (GS) and glutamine oxoglutarate aminotransferase (GOGAT) [12]. Long-term water stress severely inhibits soil N mobility, resulting in a low N uptake, negatively impacting plant growth [13]. In addition, drought stress inhibits the ability of trees to metabolize N by disrupting its metabolizing enzyme activities. The scenario mentioned above indicates that N availability is strongly related to drought since drought severely changes accessibility and mineralization.

Plant growth requires P, which accounts for approximately 0.2% of the plant’s dry weight. However, approximately 80% of the total soil-P is immobile and inaccessible [14], inhibiting growth and metabolism. Therefore, the availability and uptake of P are critical to plant metabolism. For instance, in P-depleted plants, photosynthesis is hampered by the lack of P-containing metabolites involved in the primary metabolism [15]. In addition, P nutrition is closely related to NO_3_^−^ uptake. For instance, a low P supply may reduce the NO_3_^−^ uptake, causing reductions in the plants’ growth and dry matter production. Many studies suggest that P fertilization improves plant growth and productivity under drought stress [9,16,17,18,19]. As a result of P supplementation, soil P deficiency is reduced, plants can tolerate stress better, and physiological, morphological, and biochemical processes are altered, leading to increased plant growth [17,18,19,20].

The Taklamakan is a hyper-arid desert characterized by low soil organic matter, a high pH, and reduced water content. These factors commonly result in extremely low soil-P availability. It is dominated by perennial xerophytes, which play critical roles in stabilizing the ecosystems in both oases and deserts [1]. In recent decades, soil-P depletions, or the severe P nutritional conditions of xerophytes, have frequently been noticed in desert ecosystems [21,22]. In this hyper-arid region, severely P-depleted soils are detrimental to desert vegetation growth and survival [1]. Based on the increased occurrences of future severe drought events and N-deposition, xerophytes are more likely to be affected by soil-P shortages than other ecosystem plants [21]. Still, little is known about how xerophytes react to soil-P availability. 

*Calligonum mongolicum* is a perennial shrub belonging to the family Polygonaceae. It is a popular choice for afforestation to fix and stabilize dunes. It is primarily found in the desert-oasis ecotone of the Taklamakan desert in northwestern China. Its assimilative shoots provide highly nutritious food for camels and sheep [23]. This species has been destroyed by overgrazing by local animals and excessive anthropogenic activities, which left the desert ecosystem vulnerable to drifting dunes. Consequently, the revegetation and restoration of *Calligonum* vegetation are urgently needed to protect the ecosystems of these oases against future climate change. Previously, *C. mongolicum* has been investigated for its root ecology [24], physiological responses to abiotic factors, including drought under N fertilization [25], and salinity under K supplementation [23]. 

To the best of our knowledge, no research has been published on whether P application can improve the drought tolerance of *C. mongolicum*. In arid regions, the seedling plantation has been widely used to restore vegetation, since natural restoration is difficult [26]. However, extreme environmental conditions in the hyperarid desert can severely affect the growth and metabolism of plants, particularly at the young seedling growth stages, causing a severe threat to their survival and establishment. This scenario makes planting young seedlings for vegetation restoration challenging in the hyperarid and saline and nutrients deficient (i.e., P) desert environment [1]. Therefore, investigating the impact of P supplementation on drought adaption strategies in young *C. mongolicum* seedlings will provide a theoretical framework for restoring *Calligonum* communities and protecting the fragile ecosystem of the Taklimakan desert. 

Therefore, it is imperative to investigate the impact of P application and drought on the growth of *C. mongolicum* seedlings to establish conservation strategies to mitigate the negative consequences of future climate change. 

In this study, we aimed to investigate(a) how P application (+P/−P) affects antioxidant mechanisms, N metabolism, and mineral nutrition of young *C. mongolicum* under water-stressed (25–30% field capacity) and well-watered (70–75% field capacity) conditions; and (b) the combined factors that influence its morpho-physio-biochemical characteristics in the hyper-arid desert. We evaluated plant growth, photosynthetic pigments, nonstructural carbohydrates and osmolytes, lipid peroxidation and antioxidant enzymes, N metabolism, and mineral nutrients to answer the above questions.

## 2. Results 

Our study examined the effects of P fertilization on multiple morpho-physiological and biochemical attributes of *C. mongolicum* seedlings under well-watered and water-stressed conditions. 

### 2.1. Changes in Growth Characteristics

**Figure 1:**
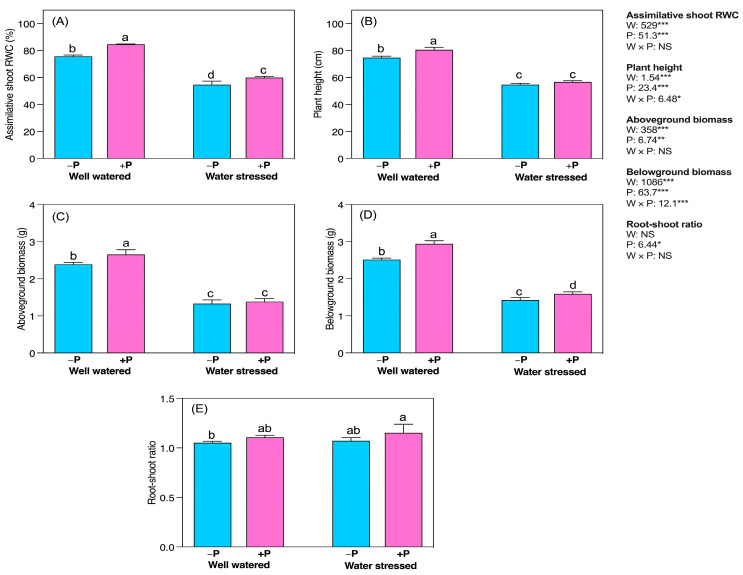
Changesin (**A**) assimilative shoots RWC, (**B**) plant height, (**C**) above-ground biomass, (**D**) below-ground biomass, and (**E**) root-shoot ratio inresponse to P supplementation, under well-watered and drought-stressed conditions. A different letter following the means indicates significant differences (*p* < 0.05) between the four treatments, as determined by Duncan’s test. Bars represent means ± SD. An overview of the two-way ANOVA assessing the effect of water (W) and phosphorus (P) treatments is presented in the right-hand corner of each panel. *, ** and *** indicate that *F* values were significant at *p* ≤ 0.05, 0.01, and 0.001, respectively.

We observed obvious changes in morphological traits under different levels of water and P fertilization (Figure 1A–E). Water-stressed plants displayed significant decreases in assimilative shoots relative water content (RWC; 30.95%), plant height (26.73%), and above- and below-ground biomass (44.29% and 43.31%, respectively), compared to well-watered plants, regardless of P fertilization (Figure 1A–D). In contrast, P fertilization caused a significant increment in assimilative shoots RWC under both water levels, compared to their unfertilized peers (Figure 1A). In water-stressed seedlings, P fertilization significantly increasedbelow-ground biomass (11.68%), as compared to their unfertilized counterparts (Figure 1D). However, P application had no significant impact on plant height and above-ground biomass under water-stressed conditions. On the contrary, P fertilization under well-watered conditions significantly increased plant height (7.81%) and above- and below-ground biomass (11.14% and 17.22%, respectively) (Figure 1B–D).

### 2.2. Changes in Chlorophyll a and Chlorophyll b Pigments

**Figure 2:**
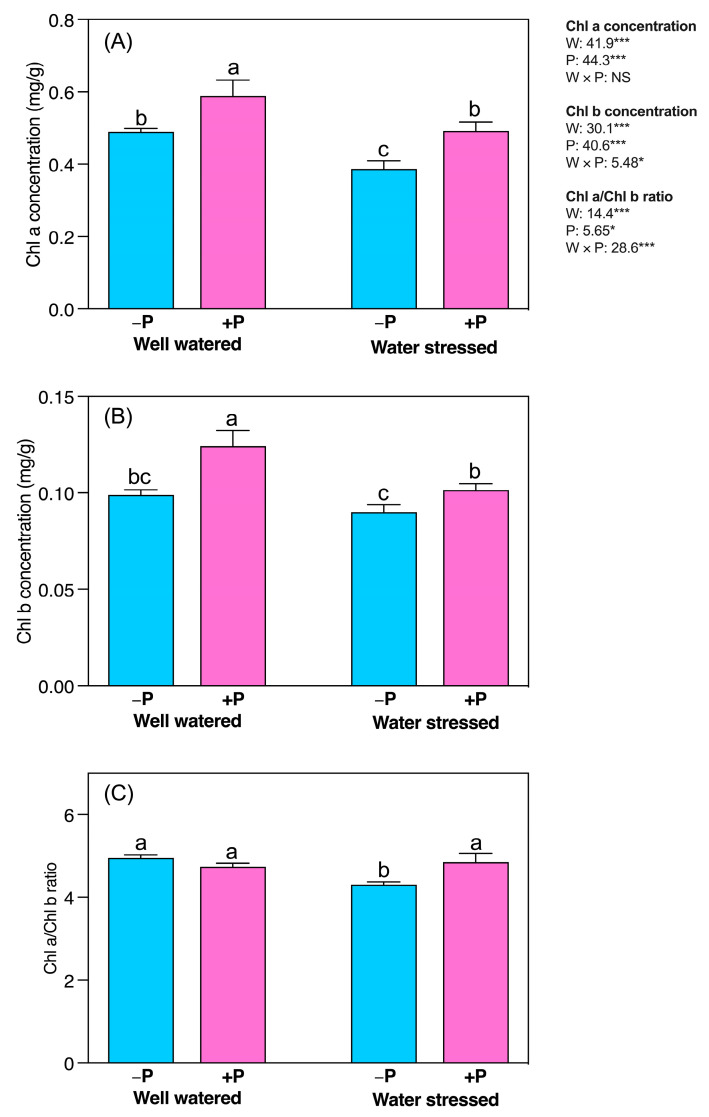
Changes in concentrations of (**A**) chlorophyll *a*, (**B**) chlorophyll *b* and (**C**) chlorophyll *a*/*b* ratio in response to P supplementation, under well-watered and drought-stressed conditions. A different letter following the means indicates significant differences (*p* < 0.05) between the four treatments, as determined by Duncan’s test. Bars represent means ± SD. An overview of the two-way ANOVA assessing the effect of water (W) and phosphorus (P) treatments is presented in the right-hand corner of each panel. * and *** indicate that *F* values were significant at *p* ≤ 0.05, 0.01, and 0.001, respectively.

Drought conditions significantly decreased the concentrations of chlorophyll *a* (Chl*a*) and chlorophyll *b* (Chl*b*) (20.87% and 9.10%, respectively) compared to well-watered conditions, regardless of P fertilization (Figure 2A,B). However, P fertilization more significantly increased the concentrations of Chl*a* (27.07%) and Chl*b* (12.79%) in water stress fertilized seedlings than in unfertilized seedlings. Chl*a* and Chl*b* increased by 20.21% and 25.48% more in well-watered seedlings treated with P fertilizer than those left unfertilized (Figure 2A,B). In addition, Chl*a* and Chl*b* significantly decreased in water-stressed (13.05%) compared to well-watered conditions. However, P fertilization significantly increased Chl*a/b* ratio (12.74%) in water-stressed seedlings compared to their unfertilized counterparts (Figure 2C). 

### 2.3. Biochemical Changes

**Figure 3:**
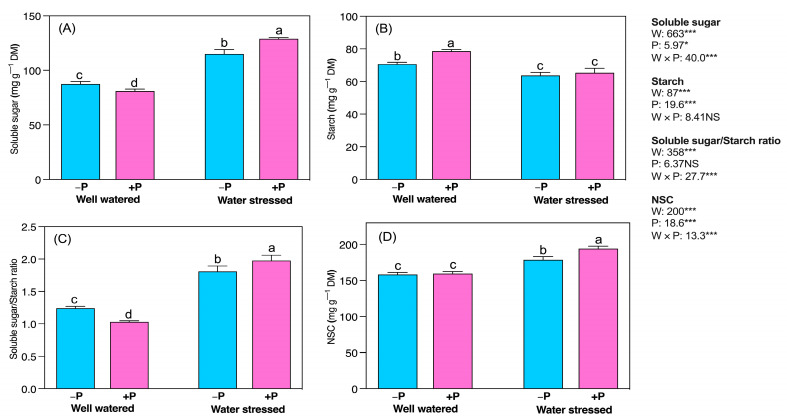
Changes inconcentrations of (**A**) soluble sugar, (**B**) starch, (**C**) soluble sugar/starch ratio, and (**D**) nonstructural carbohydrates in response to P supplementation under well-watered and drought-stressed conditions. A different letter following the means indicates significant differences (*p* < 0.05) between the four treatments, as determined by Duncan’s test. Bars represent means ± SD. An overview of the two-way ANOVA assessing the effect of water (W) and phosphorus (P) treatments is presented in the right-hand corner of each panel. * and *** indicate that *F* values were significant at *p* ≤ 0.05, 0.01, and 0.001, respectively.

Soluble sugar and starch concentrations exhibited obvious changes in response to different water and P fertilization levels (Figure 3A,B). Drought significantly increased soluble sugar (31.31%) but decreased starch (9.90%) compared to well-watered conditions, regardless of P fertilization (Figure 3A,B). P fertilization exhibited the opposite effect on soluble sugar and starch in well-watered seedlings:decreased soluble sugar (7.62%) andincreased starch (11.29%), compared with their unfertilized counterparts. However, P fertilization significantly increased soluble sugar (12.02%), but the starch concentration remained unchanged in water-stressed seedlings compared to their unfertilized counterparts (Figure 3A,B).

**Figure 4:**
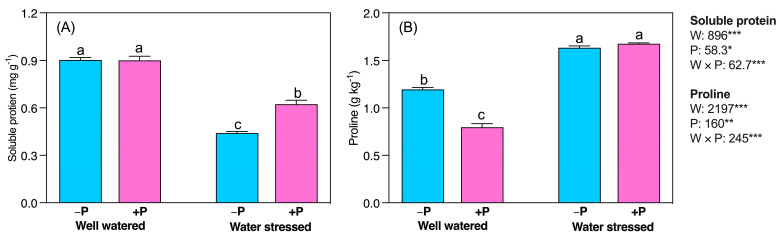
Changes inconcentrations of (**A**) soluble protein and (**B**) proline in response to P supplementation, under well-watered and drought-stressed conditions. A different letter following the means indicates significant differences (*p* < 0.05) between the four treatments, as determined by Duncan’s test. Bars represent means ± SD. An overview of the two-way ANOVA assessing the effect of water (W) and phosphorus (P) treatments is presented in the right-hand corner of each panel. *, ** and *** indicate that *F* values were significant at *p* ≤ 0.05, 0.01, and 0.001, respectively.

The ratio of soluble sugar to starch (SS/ST) significantly increased (45.82%) in water-stressed seedlings compared to well-watered seedlings, regardless of P fertilization (Figure 3C). However, SS/ST significantly decreased in well-watered (16.99%) but increased in water-stressed seedlings (9.23%) under P fertilization (Figure 3C). Nonstructural carbohydrates (NSCs) were more significantly increased in water-stressed seedlings (12.91%) than in well-watered seedlings. NSCs levels were unaffected by P fertilization in well-watered seedlings, but significantly increased in water-stressed seedlings (8.67%) (Figure 3D). Water-stressed significantly decreased the concentration of soluble protein (51.29%) but increased proline (36.82%) compared to the well-watered condition (Figure 4A). P fertilization increased soluble protein (41.66%) in water-stressed seedlings, whereas it decreased proline (33.37%) in well-watered seedlings (Figure 4A,B). 

### 2.4. Changes in ROS Accumulation and Lipid Peroxidation 

**Figure 5:**
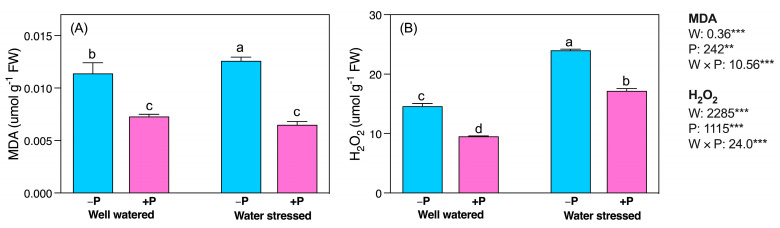
Changes in concentrations of (**A**) MDA and (**B**) H_2_O_2_ in response to P supplementation, under well-watered and drought-stressed conditions. A different letter following the means indicates significant differences (*p* < 0.05) between the four treatments, as determined by Duncan’s test. Bars represent means ± SD. An overview of the two-way ANOVA assessing the effect of water (W) and phosphorus (P) treatments is presented in the right-hand corner of each panel. ** and *** indicate that *F* values were significant at *p* ≤ 0.05, 0.01, and 0.001, respectively.

Lipid peroxidation was significantly higher in water-stressed than in well-watered seedlings. The MDA and H_2_O_2_ were significantly higher (11.11- and 64.34%, respectively) in water-stressed compared to well-watered seedlings (Figure 5A,B). However, P fertilization significantly reduced the concentration of MDA and H_2_O_2_ in well-watered (35.50% and 34.78%, respectively) and water-stressed (48.81- and 28.45%, respectively) seedlings, compared to their unfertilized counterparts (Figure 5A,B).

### 2.5. Changes in Activities of Antioxidant Enzymes

**Figure 6:**
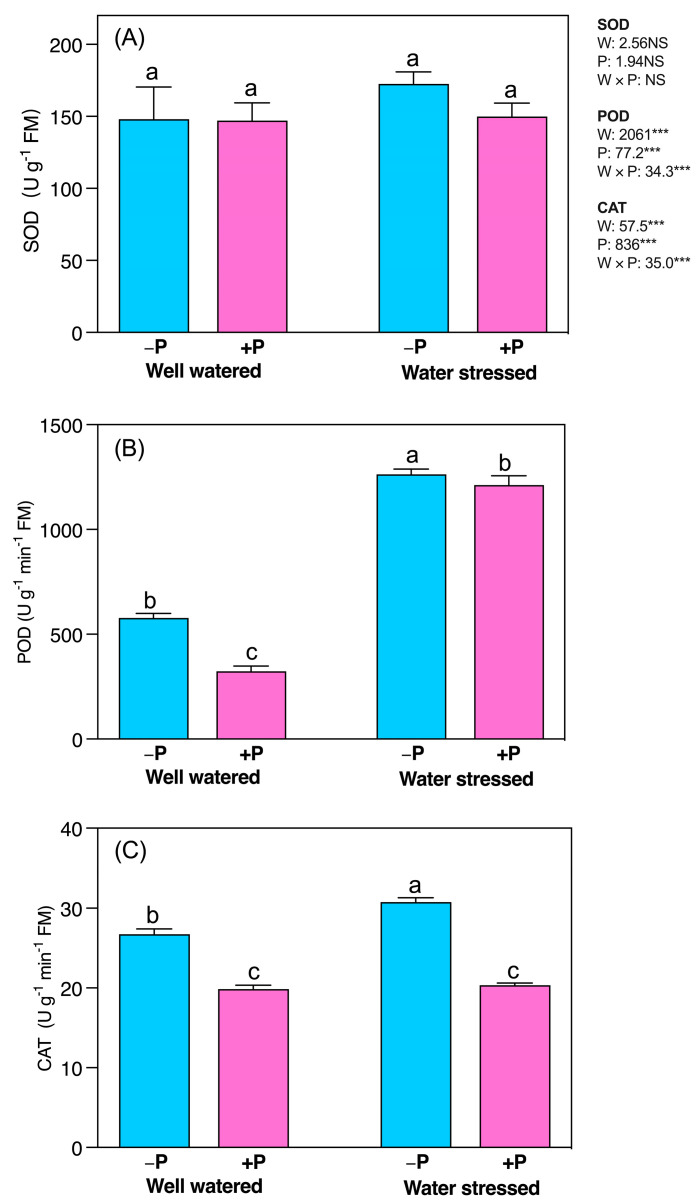
Changes in the activity of (**A**) SOD, (**B**) POD, and (**C**) CAT in response to P supplementation, under well-watered and drought-stressed conditions. A different letter following the means indicates significant differences (*p* < 0.05) between the four treatments, as determined by Duncan’s test. Bars represent means ± SD. An overview of the two-way ANOVA assessing the effect of water (W) and phosphorus (P) treatments is presented in the right-hand corner of each panel. *** indicate that *F* values were significant at *p* ≤ 0.05, 0.01, and 0.001, respectively.

Water stress significantly increased the activities of POD (118.61%) and CAT (15.12%), compared to a well-watered regime, regardless of P fertilization (Figure 6A–C). P fertilization had no significant effects on SOD under both water regimes and POD under water-stressed conditions. P fertilization significantly reduced POD (43.93%) in well-watered and CAT in well-watered and water-stressed seedlings (25.75% and 33.89%, respectively), compared to unfertilized seedlings (Figure 6B,C).

### 2.6. Changes in Enzymatic Activities of Nitrate Assimilation

**Figure 7:**
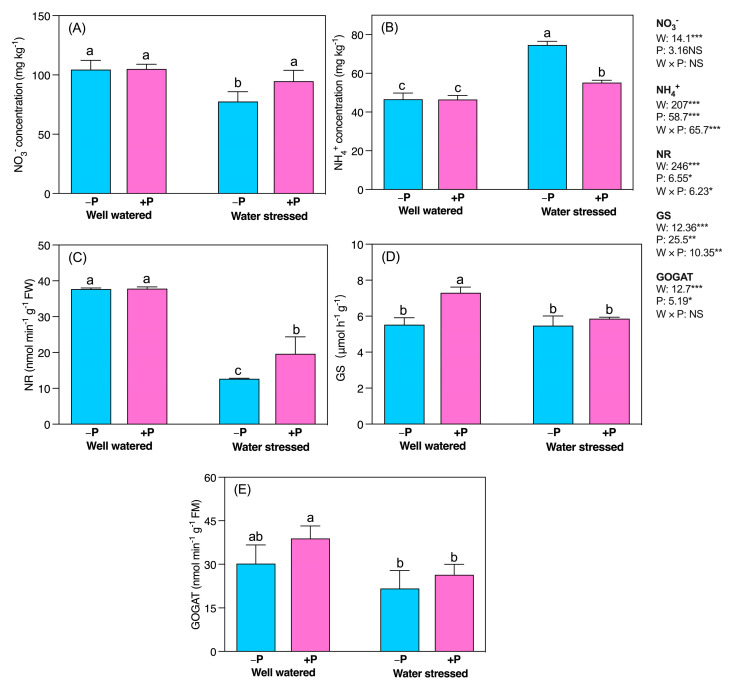
Changes in concentrations of (**A**) NO_3_^−^ and (**B**) NH_4_^+^**,** and enzymatic activity of (**C**) NR (**D**) GS, and (**E**) GOGAT in response to P supplementation, under well-watered and drought-stressed conditions. A different letter following the means indicates significant differences (*p* < 0.05) between the four treatments, as determined by Duncan’s test. Bars represent means ± SD. An overview of the two-way ANOVA assessing the effect of water (W) and phosphorus (P) treatments is presented in the right-hand corner of each panel. *, ** and *** indicate that *F* values were significant at *p* ≤ 0.05, 0.01, and 0.001, respectively.

Water stress significantly decreased the NO_3_^−^ (25.74%) concentration compared to well-watered conditions. In contrast, NH_4_^+^ significantly increased under water-stressed conditions, regardless of P fertilization. Further, P fertilization had no significant effect on NO_3_^−^ and NH_4_^+^ under well-watered conditions. Under water-stressed conditions, P fertilization significantly enhanced the NO_3_^−^(22.05%) but reduced the NH_4_^+^ (21.56%) concentration (Figure 7A,B), compared with their unfertilized counterparts. Further, waterstress significantly reduced nitrate reductase (NR) activity (66.46%) compared to well-watered conditions. P fertilization significantly increased NR activity in water-stressed conditions (55.08%). Still, it caused no significant increase under well-watered conditions, compared with their unfertilized counterparts (Figure 7C). Water stress had no significant effect on GS and GOGAT activities, regardless of P fertilization, as compared to well-watered conditions. However, P fertilization significantly increased GS activity in well-watered seedlings by 32.02%, compared with their unfertilized counterparts (Figure 7D). Further, P fertilization had no significant effect on GS activity under water-stressed and GOGAT activity under both water levels, compared with their unfertilized counterparts (Figure 7E).

### 2.7. Changes in Mineral Nutrients

**Figure 8:**
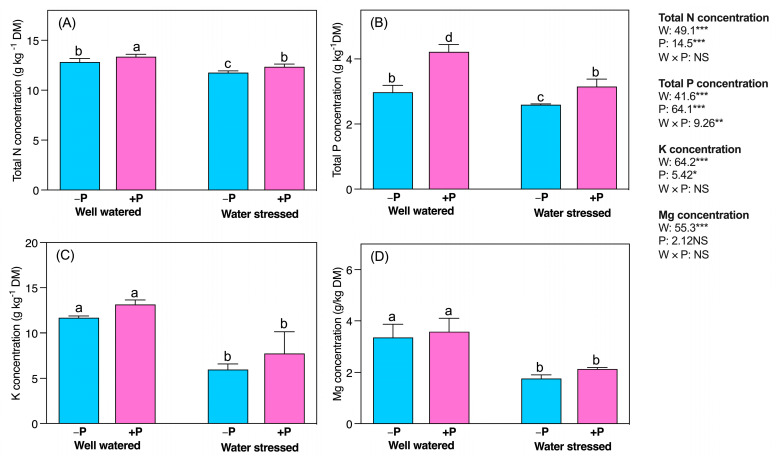
Changes in the concentrations of (**A**) TN, (**B**) TP, (**C**) K, and (**D**) Mg in response to P supplementation, under well-watered and drought-stressed conditions. A different letter following the means indicates significant differences (*p* < 0.05) between the four treatments, as determined by Duncan’s test. Bars represent means ± SD. An overview of the two-way ANOVA assessing the effect of water (W) and phosphorus (P) treatments is presented in the right-hand corner of each panel. *, ** and *** indicate that *F* values were significant at *p* ≤ 0.05, 0.01, and 0.001, respectively.

Waterstress significantly altered the concentration of mineral nutrients. N, P, K, and Mg concentrations significantly reduced (8.30-, 12.87-, 48.71-, and 47.53%, respectively) in water-stressed seedlings, compared to well-watered, regardless of P fertilization (Figure 8A–D). In both well-watered and water-stressed conditions, P fertilization significantly increased the concentrations of N (4.225% and 5%, respectively) and P (41.77% and 21.53%, respectively), compared to their unfertilized counterparts (Figure 8A,B). Contrarily, P fertilization did not cause significant changes in K and Mg concentrations, compared with their unfertilized counterparts, regardless of water availability (Figure 8C,D). 

### 2.8. Relationship between the Studied Parameters

**Figure 9:**
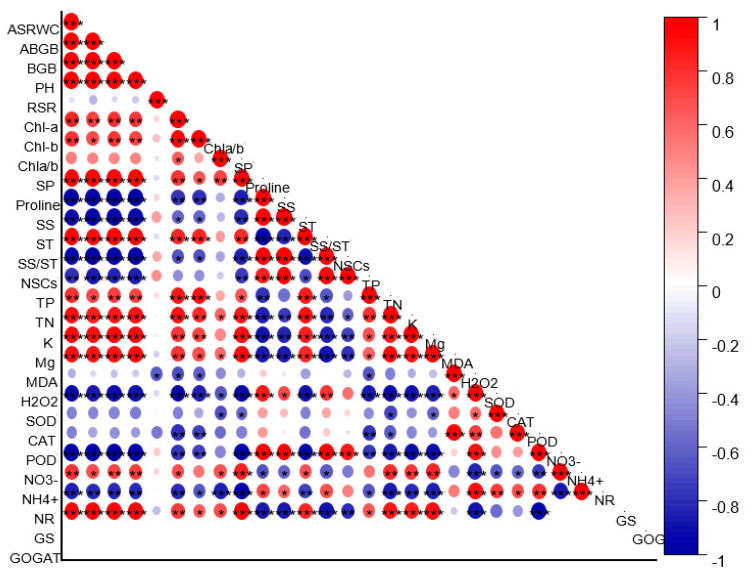
Pearson correlation analysis between study parameters. Assimilative shoots RWC (relative water content), ABGB (above-ground biomass), BGB (below-ground biomass), PH (plant height), RSR (root-shoot ratio), Chl−*a* (chlorophyll *a*), Chl−*b* chlorophyll *b*, Chla/*b* (chlorophyll *a*/*b* ratio), SP (soluble protein), proline SS (soluble sugar), ST (starch), SS/ST (soluble sugar/starch ratio), NSCs (nonstructural carbohydrates), TP (Total P), TN (total N), K (potassium), Mg (magnesium), MDA (malondialdehyde), H_2_O_2_, (hydrogen peroxide), SOD (superoxide dismutase),CAT (catalase), POD (peroxidase), NO_3_^−^ (nitrate), NH_4_^+^ (ammonium), NR (nitrate reductase), GS (glutamine synthetase), GOGAT (glutamine oxoglutarate aminotransferase). * *p* ≤ 0.05, ** *p* ≤ 0.01, *** *p* ≤ 0.001.

The correlation analysis revealed a strongnegative correlation of proline, H_2_O_2_, SS, SS/ST, NSCs, POD, and NH_4_^+^ with assimilative shoots RWC, ABGB (above-groundbiomass), BGB (below-groundbiomass), PH (plantheight), Chl-*a* and Chl-*b*, and SP (soluble protein) (Figure 9). The NR, GS, and GOGAT exhibited a strongpositive correlation with assimilative shoots RWC, ABGB, BGB, PH, Chl-*a* and Chl-*b*, SP, and ST (starch). At the same time, theirnegative correlation was observedwithproline, SS (solublesugar), SS/ST, NSCs, MDA, H_2_O_2_, SOD, POD, CAT, and NH_4_^+^ (Figure 9).

### 2.9. Results Summary

We observed a positive interaction between water and P supply on the most studied variables related to plant fitness (below-ground biomass, height, chlorophyll, and NH_4_^+^ content) and storing (starch) resources (Table S1). In contrast, we observed a negative interaction between water and P supply in the most studied variables related to stress avoidance (CAT, POD, soluble sugars, proline) and H_2_O_2_ production (Table S1). Thus, the interaction analyses strongly suggest that a positive synergy enhances growth capacity and resource production when water and phosphorus are enhanced. Contrarily, when both resources are supplied together, there exists a synergy decreasing plant stress.

## 3. Discussion

Our findings demonstrated that *C. mongolicum* was adversely affected by drought in terms of reduced assimilative shoots RWC, shoot and root biomass, chlorophyll pigments, mineral nutrition, N metabolism, and ROS and MDA concentrations. However, P fertilization contributed positively to the growth and metabolism of *C. mongolicum*, which indicates that it requires high levels of P during the seeding stage of its growth.

### 3.1. P Fertilization Improvedthe Growth and Biomass of Young Seedlings

Our study found that plant height and above- and below-ground biomass was significantly decreased in water-stressed compared to well-watered seedlings, suggesting that *C. mongolicum* is highly sensitive to soil water shortages. When the soil available-P concentration cannot meet the demands of plant growth, plants can respond to the P deficiency by reducing biomass accumulation [27]. Our findings are consistent with previous studies on different plant species [9,20,28,29]. 

However, P fertilization resulted in a significant increase in the height and biomass under well-watered conditions, suggesting that *C. mongolicum* has high P demands and that the soil in the hyper-arid desert lacks enough P to meet the plant’s functional requirements [30]. These findings are consistent with previous studies reporting increased growth rates and biomass accumulation in plants fertilized with P under moist soil conditions [9,20,31]. However, P application had no significant impact on plant height and above-ground biomass under water-stressed conditions. In contrast, P fertilization significantly increased assimilative shoots RWC and below-ground biomass; however, the percentage increase was lower than the effect of P fertilization under well-watered conditions (9.86 < 12.19% and 11.68 < 17.22%, respectively). This scenario indicates that P mobility strongly decreases in water deficit conditions [32]. Previous studies exhibited that fertilization on plant growth gradually decreases with decreases in soil moisture and even disappears completely under extremely dry conditions [33]. 

Further, we observed that P fertilization resulted in an increased root-shoot ratio of water-stressed seedlings compared to well-watered seedlings. Higher root biomass contributes to drought tolerance by increasing the roots’ ability to extract soil moisture [34,35]. We suggest that P fertilization enhances the growth and root biomass of young water-stressed *C. mongolicum,* thereby increasing the shrub’s drought tolerance in a hyper-arid, P-deficient soil condition. In hyperarid desert conditions, xerophytes require a large root system to quickly exploit groundwater resources, reduce the risk of water and nutrient deficiencies, and ensure their survival [36].

### 3.2. Improved Assimilative Shoots Relative Water Content (RWC)

The relative water content (RWC) of leaves reflects the balance between the water supply to the leaves and the transpiration rate. Therefore, it is considered an important indication of the water status of plants [37]. In our study, assimilative shoots RWC significantly decreased under water-stressedconditions compared with the well-watered condition. A reduction in leaf RWC under water-stressed conditions has also been observed in various plant species [9,17,18,38], including *C. mongolicum* [25]. P addition has been shown to increase plants’ leaf relative water content (LRWC) in water-stressed plants [39,40]. Likewise, our study exhibits that P fertilization significantly increased the assimilative shoots RWC of *C. mongolicum,* regardless of water availability. We speculate that the P application may have allowed *C. mongolicum* to accumulate more osmolytes, compared to unfertilized plants [9]. The higher concentrations of osmolytes could have altered the osmotic potential and facilitated water movement in the assimilative shoots. In previous studies, increased plant RWC has been attributed to either the improvement in the root’s ability to absorb water or nutrients or an improved ability of the plant’s tissues to conserve water [17,40].

### 3.3. P Fertilization Improved Photosynthetic Chlorophyll Pigments 

In our study, photosynthetic pigment concentrations (Chl*a* and Chl*b*) were significantly lower in water-stressed than well-watered seedlings, regardless of the P application. This scenario indicates that oxidative stress damages photosynthetic pigments in water-stressed seedlings. Previous studies demonstrated that Chl*a* and Chl*b* are sensitive to soil water shortage [9,17,31,38] and reported that drought stress could damage photosynthetic pigments. A previous study [41] demonstrated that soil dehydration damages the lamellae vesiculation and chloroplast membranes, which results in decreased chlorophyll concentration. Therefore, we suggest that a water deficit condition had caused the degradation of photosynthetic pigments, leading to the diminished photosynthetic capacity and growth of *C. mongolicum* seedlings. 

P fertilization significantly increased the concentrations of chlorophyll pigments in *C. mongolicum* under both water regimes. Previous studies reported that P fertilization increases photosynthetic pigment synthesis in plants during drought, corroborating our findings [9,38]. In water-stressed plants, improved photosynthetic pigments have been shown to increase light harvestingefficiency, thus resulting in a higher net photosynthetic rate (Pn) [9]. In contrast, someother P fertilization studies reported a negligibleimprovementin chlorophyll concentrations of water-stressed plants due to variations in experimental duration, levels and durations of water deficits, and the drought tolerance ability of a particular plant species [42,43]. Although, our results are consistent with previous studies, which also suggest that the application of P improves photosynthetic pigment synthesis in water-stressed plants [19,20,38].

### 3.4. P Fertilization Increased Soluble Protein, and Carbohydrate Concentrations

We found that the soluble protein concentration was lower in water-stressed than in well-watered seedlings. Some potential explanations for this result include an increase in protease enzyme activity, increased proteolysis, or reduced protein synthesis, along with a lower photosynthetic rate (i.e., less carbon to build metabolites), in water-stressed plants. Water stress significantly increased the proline concentration compared to well-watered conditions. A high proline accumulation may reflect osmotic adjustments under drought stress and aid in ROS detoxification, contributing to membrane and macromolecule protection [44]. It has been reported that proline accumulates under low moisture conditions due to a reciprocal mechanism involving the activation of enzymes required for its synthesis and the suppression of enzymes required for its degradation [45]. Nonstructural carbohydrates (NSCs), such as soluble sugars and starches, are the products of plant photosynthesis. NSCs indicate the amount of substance needed for plant survival, growth, and buffering capacity under adverse environmental conditions [46] and therefore play a critical role in regulating plant physiological phenomena in response to stress conditions. In our study, NSCs concentrations in assimilative shoots varied significantly in response to water and P fertilization, which could be attributed to the physiological mechanisms and the changes in the NSC components. We observed a lower starch concentration and higher concentrations of soluble sugar, NSCs, and soluble sugar/starch ratios in water-stressed seedlings than in well-watered seedlings, irrespective of P treatment. Drought stress causes stomatal closure and retrains photosynthetic C gain, forcing the plants to use starch reserves for metabolic requirements [8,47]. Likewise, our study demonstrated that water-stressed *C. mongolicum* maintained a high ratio of soluble sugar to starch in assimilative shoots, regardless of P application. Therefore, water-induced increases in soluble sugar and reductions in starch within the assimilation shoots of *C. mongolicum* may be explained by converting stored starch into soluble sugars [48] for osmotic purposes adjustment. These results are not surprising, since plants in hyper-arid desert conditions (water deficit) require more soluble sugars for metabolism, since water deficit inhibits the important process of photosynthesis and reduces CO_2_ assimilation but does not substantially reduce respiration rates [49]. A sufficient concentration of soluble sugars is essential for osmotic adjustment for optimal phloem turgor to maintain phloem transport and refill xylem embolism when there is a water shortage [50]. 

P fertilization has been reported to play a critical role in regulating carbohydrate concentrations under drought stress conditions by altering the activity of correlative enzymes in carbohydrate metabolism [18,51]. In our study, soluble sugar levels were significantly higher in P-fertilized seedlings than in non-fertilized seedlings, which is consistent with previous findings [9,38]. 

### 3.5. P Fertilization Reduced Lipid Peroxidation, ROS Production, and Antioxidant System 

Drought stress often induces the formation of ROS, including anion radicals (O_2_^−^) and hydrogen peroxide (H_2_O_2_), which react with lipids, proteins, and DNA and lead to oxidative damage and the disrupting of normal cell function and a serious reduction in plant growth [52,53]. In our study, there was a significant increase in H_2_O_2_ concentrations in water-stressed seedlings, regardless of P fertilization, which may reflect a decrease in photosynthetic rate and other physiological changes occurring due to drought stress, seemingly corroborated by correlation analysis (Figure 9). The elevated ROS production resulted in significant membrane lipid peroxidation damage, as demonstrated by the significantly increased MDA concentration in the water-stressed *C. mongolicum* seedlings compared to the well-watered seedlings. Excessive ROS also damage protein structures and inactivate enzymes that function in various physiological processes [54]. 

Plant antioxidant enzymes, such as SOD, POD, and CAT, are essential in maintaining a dynamic equilibrium by scavenging excess ROS. The antioxidant enzyme activity of plants can be correlated directly with the ability of the plant to scavenge ROS (Figure 9). Our study showed that water-stressed seedlings had significantly higher enzyme activities (SOD, POD, and CAT) than well-watered seedlings, regardless of P fertilization. Similar results were also reported in other studies conducted on various plant species [9,55]. 

P fertilization did not change SOD activity under both water regimes and significantly decreased POD activity under water-stressed conditions, whereas CAT was significantly reduced in both water regimes, compared to non-fertilized seedlings. We suggest that *C. mongolicum* significantly increase POD and CAT activities to scavenge ROS in response to water stress. In contrast, applying P helps to prevent ROS-induced damage, as demonstrated by reduced MDA and improvements in other physiological functions.

### 3.6. P Fertilization Increased N-Metabolism by Increasing Its Assimilative Enzymes 

Plants readily absorb and utilize two forms of soil N (NO_3_^−^ and NH_4_^+^) in their growth. Drought stress has been associated with changes in the metabolism of N by altering the activity of N metabolic enzymes in previous studies [56,57]. Changing enzyme activity can significantly affect the efficiency of N uptake and utilization. In our study, water stress significantly declined NO_3_^−^ concentration, compared to well-watered conditions. NO_3_^−^ promotes plant growth, osmotic regulation, and assists plants in adapting to abiotic stress conditions [58]. It is also crucial for stomatal closure by affecting the depolarization of guard cells [59]. 

Nitrate reductase (NR) activity was more significantly reduced in water-stressed conditionsthan in well-watered conditions. The reduction in NR might be attributed to a reduction in leaf photosynthesis, resulting from stomatal closure, which inhibits growth. A previous study [60] reported significant reductions in the activity of NR and NiR enzymes and the expression levels of GmNR and GmNiR in soybean leaves leading to impaired nitrogen assimilation. Several studies have claimed that drought stress decreases NR activity [61,62,63]. Decreasing NR activity may have some advantages for plants since it contributes rapidly to maintaining osmotic pressure in photosynthetic cells through an increase in the NO_3_^−^ concentration.

NH_4_^+^ is assimilated by glutamine synthase (GS) and glutamine oxoglutarate aminotransferase (GOGAT). The GS/GOGAT metabolism cycle is inhibited by drought stress, which weakens NH_4_^+^ assimilation, causing cell death and toxicity [58,60]. In our study, NH_4_^+^ significantly increased under water-stressed, compared to well-watered conditions, regardless of P fertilization. Further, water stress did not significantly affect GS/GOGAT metabolism in our study (Figure 7D,E), indicating that *C. mongolicum* seedlings accelerated NH_4_^+^ to withstand drought stress. A recent study demonstrated that GS activity remained unchanged in *S. japonica* seedlings under drought stress, while GOGAT activity increased in both roots and leaves [62]. Furthermore, previous studies have demonstrated that P application can adaptively regulate N metabolism under water stress by modulating the activity of its associated enzymes [18,57,63]. We observed that P fertilization increased the NR activity under drought stress, whereas there was no significant effect on NR under well-watered and GS/GOGAT activities under both stress conditions, compared with their unfertilized counterparts.

NH_4_^+^ is not only a vital component of living cells, but also an essential intermediate in plant metabolism. However, it can adversely affect the metabolism of higher plants when present in excess [64]. In our study P fertilization significantly reduced NH_4_^+^ in water-stressed conditions (Figure 7B), compared with their unfertilized counterparts. Therefore, we suggest that P fertilization might have decreased or prevented toxic effects associated with the excessive NH_4_^+^ accumulation under water stress [18]. A previous study [18] reported that glutamate dehydrogenase (GDH) activity under P-fertilized water-stressed seedlings was higher than in well-watered conditions. We propose that P fertilization under waterstress might have increased the GDH activity, which had accompanied the GS/GOGAT cycle within *C. mongolicum*, for detoxifying excess NH_4_^+^. 

### 3.7. P Fertilization Improved Mineral Nutrients 

We observed significant reductions in the concentration of assimilating shoot N, P, K, and Mg in water-stressed seedlings, compared to well-watered. Water availability plays a key role in nutrient uptake and assimilation, and several studies demonstrated that decreased soil moisture reduces mineral nutrient uptake and concentrations [28,65], which corroborates our findings. Compared to their unfertilized counterparts, P fertilization resulted in significant increases in the assimilation of shoot N and P, while it did not significantly affect the concentrations of K and Mg. Previous studies reported that P fertilization increases plant N and P concentrations [66,67]. Both N and P contribute to the process of photosynthesis, cell growth and metabolism, and protein synthesis [68]. Hence, we suggest that a P-fertilization-induced increase in shoot N and P concentrations might have improved photosynthesis and protein synthesis, resulting in the improved growth of *C. mongolicum*, compared with their unfertilized counterparts. 

## 4. Materials and Methods

### 4.1. Experiment Design 

The present study was conducted between June and early October 2021 in an outdoor nursery at the Cele National Station of Observation and Research for Desert-Grassland Ecosystem (37°00′56″ N, 80°43′81″ E), Chinese Academy of Sciences, located at the southern edge of the Taklamakan Desert, Northwest China. This region is extremely hyper-arid, with a mean yearly temperature and precipitation of 11.9 °C and 35 mm, respectively. At least eight seeds collected from the natural desert were sown in each 25 L pot (ca. 18%), filled with 20 kg of homogenized soil (aeolian loamy sand with organic C, 2.99 g kg^−1^; total N, 0.23 g kg^−1^; total P, 0.60 g kg^−1^ and total K, 23.11 g kg^−1^). The pots were arranged in a complete randomized design (RCD) and watered regularly. 

### 4.2. Treatments Application

Following the seedlings’ establishment under regular management for one month, they were thinned to one healthy and uniform seedling per pot and exposed to four treatments for three months: two levels of water treatments (well-watered [control 70–75% field capacity] and water-stressed [severe drought 25–30% field capacity]) and two rates of P fertilization (+P and −P). There were 12 replications of each treatment. We calculated the soil relative water content (SRWC) for the two water treatments using a weighted method [9]. We applied sodium di-hydrogen phosphate (NaH_2_PO_4_, 25.5% P) as a P fertilizer, with the dose consisting of 390 mg P mixed in 400 mL of water per pot. Five rounds of fertilization were carried outafter every 20 days. Besides, Pots were rotated once a week throughout the experiment to avoid the possibility of systematic errors associated with variations in environmental conditions. At the end of the experiment, all of the plants were harvested. Shoot samples are immediately put in Ziploc bags and stored at −80 °C for physio-biochemical determination in the laboratory.

### 4.3. Growth Parameters

#### 4.3.1. Determination of Assimilative Shoot Relative Water Content 

A single sample of assimilative shoots from each plant was collected, and their fresh weight (FW) was determined. We immediately dipped the samples into distilled water at a temperature of 4 °C for 4 h in the dark to measure the turgid weight (TW). To determine the dry weight (DW), the samples were placed in a 75 °C oven for 24 h. The assimilative shoot relative water content (LRWC) was calculated by using the following equation:

Assimilative shoot RWC = [(FW − DW)∕(TW − DW)] × 100%

#### 4.3.2. Measurement of Plant Growth and Biomass 

The plant height (cm) was measured using a measuring tape. The above- and below-ground plant parts were separated and weighed after being removed from the soil. We calculated R/S (root-shoot) ratios by dividing the biomass of the plants’ above- and below-ground parts by the ratio of their dry weights.

### 4.4. Physio-Biochemical Analysis

#### 4.4.1. Measurement of Photosynthetic Pigments

Fresh assimilating shoots were immersed in 95% ethanol (*v*/*v*) to extract the photosynthetic chlorophyll pigments. We determined the amount of chlorophyll a (Chl*a*) and chlorophyll b (Chl*b*) by reading the absorbance at 665 nm and 649 nm, respectively [13], using a spectrophotometer (Type UV-754; Shanghai Accurate Scientific Instrument Co., Shanghai, China). The chlorophyll concentrations were determined using the following equations (mg g^−1^ FM).

Chl*a* = 13.98 A665 − 6.88 A649 (1)

Chl*b* = 24.96 A649 − 7.32 A665 (2)

Chl*a*/*b* = Chl *a*/Chl*b* (3)

Chl = Chl*a* + Chl*b* (4)

#### 4.4.2. Determination of Nonstructural Carbohydrates

Dried shoot samples were ground into a fine powder using a ball mill. The colorimetric determination of the soluble sugar concentration was determined using glucose, following a standard method [69]. Starch was measured calorimetrically at 620 nm, with a slight modification in the anthrone method [70]. The NSC represented the sum of soluble sugars and starch contents. 

#### 4.4.3. Determination of Soluble Protein and Proline

The proline extraction (0.2 g fresh assimilative shoot) was conducted using two mL of 10% acetic acid and five mL of 3% salicylic acid, respectively. The resulting supernatants were analyzed according to a standard method [61]. Approximately 0.3 g of fresh assimilative shoots were used to determine the concentration of soluble proteins using bovine serum albumin as the standard [71]. 

#### 4.4.4. Oxidative Stress Indicators

Hydrogen peroxide (H_2_O_2_) concentrations were determined by a standard procedure [72]. The 0.2 g freshly assimilative shoots were homogenized in 5 mL of trichloroacetic acid (0.1%) in an ice bath, transferred to tubes, and centrifuged at 5000× *g* for 10 min (4 °C). Next, we centrifuged the supernatant comprising of 0.1 mL of titanium reagent (50 µL of 20% titanium tetrachloride) and 0.2 mL of ammonia, centrifuged at 10,000× *g* for 10 min. Following five washes with acetone, the precipitate was centrifuged at 10,000× *g* for 10 min, after which 3 mL of 1 M H_2_SO_4_ was added. The absorbance was read at 410 nm using a spectrophotometer (Type UV-754; Shanghai Accurate Scientific Instrument Co., Shanghai, China).

The malondialdehyde (MDA) concentration was assessed based on the thiobarbituric acid (TBA) test for the evaluation of lipid peroxidation [73]. Fresh assimilative shoot samples (0.5 g) were homogenized in 1 mL of 5% trichloroacetic acid (TCA) and centrifuged for 10 min at 5000× *g* (4°C). Using a separate tube, 4 mL of the supernatant was added to 2 mL of 20% TCA (containing 0.5% TBA), and the mixture was heated at 100 °C for 15 min before centrifugation at 5000× *g* for 10 min. Using a spectrophotometer, OD450, OD532, and OD600 nm were determined, and the concentration of MDA was calculated according to the following equation:

MDA (mol g^−1^ FW) = 6.45 (OD532 − OD600) − 0.56OD450.

#### 4.4.5. Antioxidant Enzyme Activities 

Approximately 0.5 g of fresh assimilative shoot samples were homogenized in liquid nitrogen and 10 mL of 50 mM sodium phosphate buffer (pH 7.8) containing 0.5 mmol EDTA and 0.15 mol NaCl to determine enzyme activity. After centrifuging the homogenate at 12,000× *g* at 4 °C (20 min), the supernatant was used to measure the activities of superoxide dismutase (SOD), catalase (CAT), and peroxidase (POD). 

We measured the activity of superoxide dismutase (SOD) following a standard method [74]. A reaction mixture of 3 mL was prepared using 50 mM phosphate buffer (pH 7.8), 75 mM of nitro-blue tetrazolium (NBT), 130 mM of methionine, and 2.0 mM of riboflavin, and 0.1 mL of enzyme extract. We assessed the activity of SOD by reading the absorbance at 560 nm using a spectrophotometer (Type UV-754; Shanghai Accurate Scientific Instrument Co., Shanghai, China). The unit of SOD activity was defined as the amount of enzyme required for the 50% inhibition of nitroblue tetrazolium (NBT) reduction.

Peroxidase activity (POD) was determined using a standard method [75] with slight modifications. A reaction mixture of 3 mL was prepared by mixing phosphate buffer (pH 6.0), 3 mmol peroxide L^−1^, and 0.25% (*v*/*v*) of guaiacol with the enzyme extract. Using a spectrophotometer (Type UV-754; Shanghai Accurate Scientific Instrument Co., Shanghai, China), absorbance (470 nm) was read at 1-min intervals for 3 min. The enzyme activity was expressed as U/g·min^−1^.

The Catalase (CAT) activity was evaluated following a previous method [76] with minor modifications. In brief, a 3 mL reaction mixture was prepared by mixing 0.1 mL of the enzyme extract with 100 mM phosphate buffer (pH 7.0) and 10 mM hydrogen peroxide. We used a spectrophotometer (Type UV-754; Shanghai Accurate Scientific Instrument Co., Shanghai, China), to measure the change in OD240 at 1-min intervals for 3 min. The calculation of CAT activity was carried out using the 39.4 mM^−1^·cm^−1^ extinction coefficient. The enzyme activity was expressed as U/g·min^−1^.

#### 4.4.6. Determination of N-Metabolizing Enzymes

The nitrate reductase (NR) activity was determined by homogenizing 0.2 g using 2 mL of 25 mM phosphate buffer saline (PBS, pH 8.7), which contained 10 mM cysteine and 1 mM EDTA, and then centrifuging for 20 min at 30,000× *g*. The resulting supernatant was tested for NR activity using a diazo-coupling method employing the Griess reagent [13]. 

The GS activity was determined by homogenizing them in 2 mL of 50 mM Tris-HCl buffer (pH 7.8; containing 15% glycerol, 0.1% TritonX-100, 1 mM of EDTA, and 14 mM of 2-mercaptoethanol) and centrifuging them twice at 4 °C for 10 min. After complexing with acidified ferric chloride, the supernatant was used to determine GS by forming glutamine hydroxamate using a 540 nm fluorescence measurement [13]. 

#### 4.4.7. Determination of Mineral Elements

We measured the concentrations of mineral nutrients in finely powdered, assimilative shoot samples. The concentration of N and P was determined following digestion with concentrated H_2_SO_4_ using a Kjeldahl Nitrogen Analyzer (K1160, Jinan Hanon Instruments Co. Ltd., Jinan, China) and a Thermo-Elemental inductively coupled plasma optical emission spectrometer (iCAP 6300). Assimilative shoot samples were soaked in HNO_3_and HF–HNO H_2_O_2_, respectively, and then the concentrations of K and Mg were determined [77]. 

### 4.5. Statistical Analysis

Measurements were replicated three times and sorted using Microsoft Excel 2019. SPSS version 16.0 (Chicago, IL, USA) was used to perform descriptive statistics two-way analysis of variance (ANOVA) considering water availability and P application as main factors, with two levels for each. Duncan’s multiple range tests were used to compare means at a significance level of 0.05. GraphPad Prism 8 was used to create the figure graphics. For interpretation purposes, Pearson correlation analyses were conducted using OriginPro 2019 software (OriginLabCarporation Northampton, Northampton, MA, USA) regarding the growth parameters, chlorophyll pigment concentrations in the shoots, N metabolism, osmolytes accumulation, mineral nutrition, and reactive oxygen species production rate, as well as antioxidant enzymatic activities. 

## 5. Conclusions

Drought adversely affected the growth and metabolism of *C. mongolicum,* as evidenced by a reduction in assimilative shoots RWC, shoot-root biomass, photosynthetic pigments, mineral nutrition, N metabolism, and an increase in the concentration of ROS and lipid peroxidation. Even so, the improved root-shoot ratio, osmotic solutes (soluble sugar, proline), and upregulation of antioxidant enzymes (CAT and POD) suggest that *C. mongolicum* drought tolerance strategies for maximizing water absorption and reducing oxidative damage are effective. In addition, P fertilization positively affected the growth and metabolism of *C. mongolicum,* which indicates its high P needs at the young seedling stage of development. In drought-stressed seedlings, P fertilization increased the seedling’s productivity by increasing assimilative shoots RWC, photosynthetic pigments, osmolytes, mineral nutrition, and N assimilation. In addition, P fertilization reduced the levels of H_2_O_2_ and MDA, leading to reduced POD and CAT activities under both water levels. P fertilization also positively affected the growth and metabolism under well-watered conditions, which indicated the soil’s high P depletion in the hyper-arid desert. 

Therefore, we suggest that P fertilization played a vital role in the growth and metabolism under well-watered conditions, thus helping in the maintenance of drought tolerance of *C. mongolicum*, largely through physiological rather than morphological adaptations. Under both water regimes, the P fertilization increased the below-ground biomass significantly, which may be helpful to young *C. mongolicum* plants for acquiring groundwater resources and reduce the risk of water and nutrient deficiency in the hyper-arid Taklamakan desert. The addition of water and P had synergistic positive effects on variables related to fitness and energy storing and negative effects on variables related to stress, strongly suggesting the positive impact of both factors on each other. This promising result can help improve field conditions and the management of *C. mongolicum* communities in the Taklamakan desert. However, further research is needed to elaborate on the underlying molecular mechanism operating under drought stress conditions to enhance the understanding of the drought-tolerance capacity of young *C. mongolicum*.

## Data Availability

The data presented in this study are available in the graphs provided in the manuscript and supplementary table.

## Supplementary Materials

The following supporting information can be downloaded at: https://www.mdpi.com/article/10.3390/plants11223054/s1, Table S1: Two-way factorial ANOVA.
