# Supplementary material for: Phosphorous Supplementation Alleviates Drought-Induced Physio-Biochemical Damages in Calligonum mongolicum
Source: Plants (Basel). 2022 Nov 11;11(22):3054. doi: 10.3390/plants11223054 (PMC9699272; doi:10.3390/plants11223054)
Supplement: Supplementary file 1 [file plants-11-03054-s001.zip › plants-1938498-supplementary.pdf]

Table S1. Two-way factorial ANOVA. Statistical results (F-statistic and P-value) of the two-way factorial ANOVA with water and phosphorus addition treatment and their interaction (when significant).

Dependent variables	Independent variables		
	Water	Phosphorus	Water x phosphorus
Assimilative shoot RWC	F = 529<br>*P* < 0.0001	F = 51.3<br>*P* < 0.0001	No significant
Aboveground biomass	F = 358<br>*P* < 0.0001	F = 6.741<br>*P* = 0.029	No significant
Belowground biomass	F = 1086<br>*P* < 0.0001	F = 63.7<br>*P* <0.0001	F = 12.1<br>*P* = 0.0084
Plant height	F = 830<br>*P* <0.0001	F = 23.4<br>*P* = 0.00089	F = 6.48<br>*P* = 0.034
Root:Shoot	F = 1.54<br>*P* = 0.25	F = 6.44<br>*P* = 0.032	No significant
Chl-a	F = 41.9<br>*P* = 0.00011	F = 44.3<br>*P* < 0.0001	No significant
Chl-b	F = 30.1<br>*P* = 0.00058	F = 40.6<br>*P* = 0.00022	F = 5.68<br>*P* = 0.044
Chla/b	F = 14.4<br>*P* = 0.0056	F = 5.65<br>*P* = 0.045	F = 28.6<br>*P* = 0.00069
Soluble proteins	F = 986<br>*P* < 0.0001	F = 58.3<br>*P* < 0.0001	F = 62.7<br>*P* < 0.0001
Proline	F = 2197<br>*P* < 0.0001	F = 160<br>*P* < 0.0001	F = 245<br>*P* < 0.0001
Soluble sugars	F = 663<br>*P* < 0.0001	F = 5.97<br>*P* = 0.040	F = 40.0<br>*P* = 0.00011
Starch	F = 87.0<br>*P* < 0.0001	F = 19.6<br>*P* = 0.0022	F = 8.41<br>*P* = 0.120
Soluble sugars:starch	F = 445<br>*P* < 0.0001	F = 0.374<br>*P* = 0.56	F = 27.7<br>*P* = 0.00076
Non-structural carbohydrates	F = 200<br>*P* < 0.0001	F = 18.6<br>*P* = 0.0026	F = 13.3<br>*P* = 0.0065
Total protein	F = 41.6<br>*P* = 0.0002	F = 64.1<br>*P* < 0.0001	F = 9.26<br>*P* = 0.016
Total nitrogen	F = 49.1<br>*P* < 0.0001	F = 14.5<br>*P* = 0.0042	No significant
Potassium	F = 64.2<br>*P* < 0.0001	F = 5.42<br>*P* = 0.045	No significant
Magnesium	F = 55.3<br>*P* < 0.0001	F = 2.12<br>*P* = 0.18	No significant
MDA	F = 0.36<br>*P* = 0.56	F = 242<br>*P* < 0.0001	F = 10.56<br>*P* = 0.012
H_2O_2	F = 2285<br>*P* < 0.0001	F = 1115<br>*P* < 0.0001	F = 24.0<br>*P* = 0.0012
SOD	F = 2.56<br>*P* = 0.14	F = 1.94<br>*P* = 0.20	No significant
CAT	F = 57.5<br>*P* < 0.0001	F = 836<br>*P* < 0.0001	F = 35.0<br>*P* = 0.00035
POD	F = 2061<br>*P* < 0.0001	F = 77.2<br>*P* < 0.0001	F = 34.3<br>*P* = 0.00038
NO_3^-	F = 14.1<br>*P* = 0.0045	F = 3.16<br>*P* = 0.11	No significant
NH_4^+	F = 207<br>*P* < 0.0001	F = 58.7<br>*P* < 0.0001	F = 56.7<br>*P* < 0.0001
Nitrate reductase	F = 246<br>*P* < 0.0001	F = 6.55<br>*P* = 0.034	F = 6.23<br>*P* = 0.037
Glutamine synthetase	F = 12.36<br>*P* = 0.0079	F = 25.5<br>*P* = 0.00098	F = 10.53<br>*P* = 0.012
Glutamine oxoglutarate aminotransferase	F = 12.7<br>*P* = 0.0068	F = 5.19<br>*P* = 0.048	No significant
